# Supplementary material for: Tree Physiological Variables as a Proxy of Heavy Metal and Platinum Group Elements Pollution in Urban Areas
Source: Biology (Basel). 2023 Aug 29;12(9):1180. doi: 10.3390/biology12091180 (PMC10526008; doi:10.3390/biology12091180)
Supplement: Supplementary file 1 [file biology-12-01180-s001.zip › biology-2561225-supplementary.pdf]

Table S1. Coordinates and concentrations of metals, and PGEs (ng g^{-1} except Cu, Mn and Zn in ug g^{-1}) plus physiological variables (index values) for each of the 50 trees studied in Logroño (La Rioja, Spain).

ID	X	Y	Chl	Flav	Fv/Fm	As	Cd	Cu	Hg	Mn	Ni	Pb	Pd	Rh	Sb	V	Zn	N	$\delta^{15}N$
1	543128	4702092	27	1.310	0.800	35.0	25.2	8.7	5.4	64.3	431.4	124.9	161.7	4.0	34.3	131.8	45.2	1.5	5.4
2	543303	4702090	38	1.098	0.810	38.8	14.2	10.8	12.4	86.5	454.1	160.1	253.3	5.9	36.1	127.5	77.8	1.8	6.4
3	543617	4702042	36	1.422	0.805	99.3	20.5	7.6	13.0	65.2	232.7	153.4	91.4	2.2	18.5	110.1	38.1	1.9	8.8
4	544515	4702241	34	1.924	0.718	123.1	22.5	4.0	16.5	105.0	143.1	202.2	107.2	2.5	28.5	134.7	33.3	1.2	5.2
5	544930	4702309	30	1.535	0.762	138.9	13.3	5.7	19.1	69.3	243.2	255.7	118.6	2.6	60.4	126.2	52.6	1.0	5.9
6	545517	4702282	37	1.329	0.806	181.7	90.8	11.6	17.8	42.3	390.0	409.2	184.2	4.3	98.9	154.0	149.2	1.9	8.1
7	545875	4702303	30	1.248	0.814	118.8	7.6	14.9	9.2	43.5	384.0	102.8	121.0	2.7	26.9	71.1	114.6	2.0	1.7
8	546434	4702047	33	1.386	0.797	160.7	12.0	10.2	20.3	73.1	293.4	251.4	141.2	3.1	134.5	174.2	77.5	1.8	6.5
9	546809	4702272	33	1.340	0.817	149.6	62.7	10.3	16.1	117.6	419.4	222.6	190.7	4.5	27.6	109.8	99.2	1.4	7.4
10	547133	4701775	41	1.445	0.778	145.3	48.4	8.1	17.7	120.4	339.7	271.8	127.9	3.0	163.7	153.8	58.6	1.5	8.0
11	542958	4701637	37	1.276	0.811	107.5	23.6	10.6	13.4	108.5	373.3	150.0	662.2	14.2	36.8	154.7	69.7	1.7	4.3
12	543217	4701647	33	1.293	0.790	38.8	29.8	8.8	9.2	43.8	231.8	87.7	164.7	3.4	26.2	111.3	49.1	1.6	5.8
13	543874	4701659	28	1.701	0.759	38.7	3.5	5.5	14.2	102.1	123.2	108.8	126.6	2.8	21.6	79.9	39.7	1.0	4.3
14	544242	4701816	35	0.889	0.814	181.7	64.8	7.7	25.9	50.0	361.4	408.2	134.4	3.0	166.2	224.8	57.6	1.9	6.8
15	545092	4701680	38	0.816	0.813	123.7	15.6	10.3	19.6	76.8	382.5	294.1	98.2	2.3	173.3	141.3	39.3	2.2	10.7
16	545411	4701564	41	0.653	0.815	178.0	43.9	19.8	14.1	65.5	417.3	241.9	82.4	1.9	53.2	148.0	53.6	2.6	10.1
17	545911	4701566	41	0.732	0.814	145.1	21.4	16.2	13.3	76.2	417.4	399.3	87.7	2.1	93.8	160.2	35.2	2.5	11.0
18	546342	4701680	35	0.696	0.810	115.4	45.7	10.0	23.1	175.8	1104.8	359.1	125.5	3.1	87.8	181.1	36.8	2.4	10.6
19	546855	4701641	44	0.758	0.809	149.3	15.9	7.4	27.8	122.5	604.6	387.2	105.8	2.9	108.0	223.7	31.0	2.4	10.4
20	547061	4701555	44	1.145	0.795	90.5	46.1	11.5	23.4	112.7	350.9	259.3	167.4	4.3	146.6	226.9	138.0	1.4	5.7
21	542501	4700970	36	1.468	0.808	97.8	3.4	4.5	12.6	34.7	98.4	162.6	130.8	3.1	55.8	138.4	42.2	0.8	4.8
22	543499	4701188	36	1.298	0.805	82.1	29.0	6.1	29.3	134.7	180.5	223.2	160.4	3.9	63.2	204.2	153.9	1.1	-0.5
23	544091	4701269	40	1.468	0.818	93.0	32.1	8.4	25.8	65.2	168.7	435.7	97.7	2.6	107.7	172.5	115.6	1.0	2.0
24	544265	4701162	35	1.800	0.770	35.0	18.3	5.4	19.0	67.8	148.8	256.0	63.1	1.5	89.9	123.3	70.2	1.0	-1.5
25	544831	4701302	33	0.840	0.820	135.7	43.3	12.5	13.7	90.0	643.5	234.7	78.0	1.6	87.9	161.9	60.8	2.8	12.0

26	545316	4701155	39	1.068	0.792	105.7	33.5	11.3	12.2	51.9	273.5	228.5	56.1	1.3	98.7	150.7	47.2	2.3	9.3
27	545747	4701144	39	0.623	0.822	89.3	19.2	12.2	12.7	55.1	378.4	233.8	75.5	1.6	82.0	162.3	56.7	2.6	11.6
28	546303	4701099	40	0.810	0.814	94.4	11.7	10.7	15.2	88.7	299.4	308.9	172.8	3.5	142.9	204.7	36.6	2.2	8.1
29	546759	4701328	46	1.273	0.816	105.1	26.4	11.8	12.7	61.9	336.3	230.5	61.2	1.7	98.7	170.9	66.9	2.4	12.2
30	547739	4700961	36	1.889	0.754	37.1	23.7	4.1	18.8	35.2	161.6	200.5	112.3	2.9	99.3	135.9	53.0	1.0	5.9
31	542805	4700916	45	1.170	0.814	88.2	3.4	6.4	34.7	91.3	269.5	254.0	128.1	3.0	110.2	219.7	30.9	1.4	7.2
32	543301	4700672	32	1.782	0.776	74.4	7.6	6.7	30.3	43.3	153.1	191.5	297.1	6.9	67.4	162.4	99.3	0.8	-0.4
33	543648	4700826	27	1.887	0.688	36.5	3.3	6.3	23.5	38.2	119.9	164.8	136.9	3.3	57.7	131.1	42.6	0.8	0.3
34	544237	4700455	33	1.749	0.737	37.5	14.3	4.9	15.2	38.4	88.1	144.1	90.8	2.2	66.8	112.5	54.8	0.8	2.8
35	545124	4700616	38	0.731	0.816	37.0	22.2	12.4	16.4	97.1	902.3	300.2	151.9	3.5	217.0	195.4	63.7	2.3	10.7
36	545305	4700612	24	0.904	0.830	38.1	123.9	11.3	14.3	117.7	523.8	225.6	112.3	2.6	82.2	186.8	60.0	2.2	9.9
37	545707	4700612	42	1.154	0.822	37.5	33.6	7.7	11.7	154.1	481.1	164.2	103.8	2.5	46.5	97.4	56.4	2.1	9.6
38	546161	4700992	36	0.937	0.812	107.8	19.9	9.9	19.2	86.7	701.1	266.6	77.2	1.8	54.4	141.3	37.2	2.2	8.9
39	546551	4700663	40	1.200	0.800	81.4	31.9	3.5	28.7	60.6	183.3	236.4	116.6	2.9	102.1	186.8	22.4	1.2	7.0
40	547532	4700650	35	1.570	0.797	148.4	9.2	3.1	21.6	57.7	157.1	192.6	75.5	1.8	87.9	153.9	21.0	1.4	5.6
41	543296	4700251	36	1.484	0.811	90.4	41.3	11.6	7.1	63.1	449.5	167.8	119.5	3.1	45.2	226.5	108.2	2.3	7.4
42	543466	4700204	33	1.187	0.796	38.5	29.1	13.6	11.8	47.6	365.7	135.2	197.4	5.0	61.2	167.9	91.9	2.1	5.1
43	543939	4700315	45	1.229	0.797	38.3	23.8	5.1	26.5	129.6	156.6	207.0	170.2	4.0	145.4	161.6	29.5	1.4	5.4
44	544328	4700146	37	1.056	0.812	38.2	14.2	8.5	8.6	107.3	376.9	151.4	97.1	2.2	55.0	145.3	46.3	2.0	8.1
45	544501	4700079	35	0.996	0.805	38.8	7.1	7.8	10.7	47.1	298.5	150.0	72.3	1.7	56.7	158.1	35.0	1.8	8.1
46	545187	4700147	39	1.033	0.830	91.8	24.3	14.9	7.9	87.7	561.0	94.6	274.7	6.2	33.6	86.8	46.4	2.6	9.8
47	545865	4700224	33	1.040	0.808	81.9	34.2	9.8	19.4	158.6	632.2	273.4	129.9	2.8	118.6	216.9	62.3	1.3	7.1
48	546517	4700032	31	1.714	0.725	38.5	44.5	4.3	22.3	38.3	124.9	188.5	66.2	1.6	57.9	186.5	51.1	0.9	2.6
49	547313	4700336	37	0.687	0.818	128.6	37.9	3.5	8.0	66.3	614.4	127.8	35.7	1.1	46.9	120.3	28.7	2.6	10.1
50	547350	4700228	40	1.032	0.803	132.6	18.7	11.0	12.6	112.4	519.1	306.0	105.6	2.6	199.2	181.7	47.9	2.1	7.0
